# Mitochondrial respiratory capacity in patients with acute episodes of bipolar disorder compared with clinical remission

**DOI:** 10.1192/j.eurpsy.2024.210

**Published:** 2024-08-27

**Authors:** A. Giménez-Palomo, M. Guitart-Mampel, A. Meseguer, M. Valentí, L. Bracco, H. Andreu, E. Vieta, G. Garrabou, I. Pacchiarotti

**Affiliations:** ^1^Bipolar and Depressive Disorders Unit, Hospital Clínic of Barcelona, IDIBAPS; ^2^Muscle Research and Mitochondrial Function Laboratory, IDIBAPS, Cellex, Internal Medicine Service, Hospital Clínic de Barcelona, Biomedical Research Networking Centre Consortium on Rare Diseases (CIBERER); ^3^Department of Psychiatry and Psychology, Institute of Neuroscience, Hospital Clínic de Barcelona, Barcelona, Catalonia, Spain, Barcelona, Spain; ^4^Department of Pathophysiology and Transplantation, University of Milan, 20122 Milan, Italy, Milano, Italy

## Abstract

**Introduction:**

Bipolar disorder (BD) is a chronic and recurrent disease characterized by acute mood episodes alternated with periods of euthymia. The available literature postulates that a biphasic dysregulation of mitochondrial bioenergetics might be observed in BD.

**Objectives:**

We aimed to explore differences in *in vivo* mitochondrial respiration (1) intra-individually: longitudinally within patients during an acute mood episode of BD and after clinical remission, and (2) inter-individually: between patients with BD on depressive or manic episodes and healthy controls (HC).

**Methods:**

Patients admitted to our acute psychiatric ward with a manic episode or bipolar depression were recruited. Different mitochondrial oxygen consumption rates (OCRs) were assessed during the acute episode (T0) and after clinical remission (T1) in one million of peripheral blood mononuclear cells (PBMC): Routine, Leak, ETC and Rox. They were measured as picomoles of oxygen per million cells (pmol O_2_/million). This experiment was also conducted in HC. High-resolution respirometry was performed at 37°C by polarographic oxygen sensors in a two-chamber Oxygraph-2k system. Manic and depressive symptoms were assessed using standardized psychometric scales. Oxygen consumption capacity was compared (1) intra-individually, during acute episodes and after clinical remission, and (2) inter-individually, during acute manic and depressive episodes, and in HC. Statistical analyses were performed with SPSS, GraphPad and R Statistics.

**Results:**

20 patients with BD (15 manic, 5 depressed) and 10 HC were included. A significant increase in the maximal oxygen consumption capacity (ETC) was observed in clinical remission (27.4 ± 17.4) compared to the acute episodes (21.1 ± 11.7, p = 0.001), which remained significant after subtracting Rox from the other rates (p = 0.001). At T1, patients admitted with a manic episode tended to show higher mean ETC (31.2 ± 18.7) compared with T0 (24.1 ± 12.0, p = 0.074); the tendency persisted after Rox subtraction (p = 0.076). Patients admitted with a depressive episode also showed higher ETC means in T1 (16.3 ± 3.8) compared to T0 (12.1 ± 3.4), but there were not significant differences (p = 0.231). When HC, manic and depressive patients at T0 were compared between them, significant differences were observed in ETC (H =8.5; p =0.014) and Rox (H =13.8; p = 0.001). After Rox deduction, differences in ETC remained (H =11.7; p = 0.003). Individuals with bipolar depression showed lower ETC rates (12.1 ± 3.4) than those with a manic episode (24.1 ± 12.0; t = -3.5, p = 0.003), which was also found after Rox deduction (p = 0.001).

**Conclusions:**

In both manic and depressive episodes in BD, mitochondrial respiration might be reduced and increase after clinical remission. Further studies with larger samples will allow to confirm these results and also to identify potential mitochondrial state-dependent biomarkers.

**Disclosure of Interest:**

A. Giménez-Palomo Grant / Research support from: AGP is supported by a Rio Hortega 2021 grant (CM21/00094) from the Spanish Ministry of Health financed by ISCIII and cofinanced by Fondo Social Europeo Plus (FSE+)., M. Guitart-Mampel: None Declared, A. Meseguer: None Declared, M. Valentí: None Declared, L. Bracco: None Declared, H. Andreu: None Declared, E. Vieta: None Declared, G. Garrabou: None Declared, I. Pacchiarotti: None Declared

